# Scientific commentary on: “Phosphorylated tau in the retina correlates with tau pathology in the brain in Alzheimer’s disease and primary tauopathies”

**DOI:** 10.1007/s00401-023-02656-z

**Published:** 2024-02-03

**Authors:** Frederike C. Oertel, Daniel Casillas, Yann Cobigo, Shivany Condor Montes, Hilary W. Heuer, Makenna Chapman, Alexandra Beaudry-Richard, Henriette Reinsberg, Ahmed Abdelhak, Christian Cordano, Bradley F. Boeve, Bradford C. Dickerson, Murray Grossman, Edward Huey, David J. Irwin, Irene Litvan, Alexander Pantelyat, M. Carmela Tartaglia, Lawren Vandevrede, Adam Boxer, Ari J. Green

**Affiliations:** 1 Weill Institute for Neurosciences, Department of Neurology, University of California San Francisco (UCSF), 675 Nelson Rising Lane, Sandler Neurosciences Building, San Francisco, CA USA; 2 UCSF Memory and Aging Center, San Francisco, CA USA; 3 Mayo Center for Sleep Medicine, Departments of Neurology and Medicine, Mayo Clinic College of Medicine and Science, Rochester, MN USA; 4 Department of Neurology, Massachusetts General Hospital and Harvard Medical School, Boston, MA USA; 5 Department of Neurology, Perelman School of Medicine, University of Pennsylvania, Philadelphia, PA USA; 6 Center for Neurodegenerative Disease Research, Perelman School of Medicine, University of Pennsylvania, Philadelphia, PA USA; 7 Taub Institute for Research on Alzheimer’s Disease and the Aging Brain, Columbia University Medical Center, New York, NY USA; 8 Department of Neurology, Columbia University Medical Center, New York, NY USA; 9 Department of Psychiatry and New York Psychiatric Institute, Columbia University Medical Center, New York, USA; 10 Department of Neurosciences, University of California San Diego, San Diego, CA 92093 USA; 11 Department of Neurology, School of Medicine, Johns Hopkins University, Baltimore, MD USA; 12 Tanz Centre for Research in Neurodegenerative Diseases, University of Toronto, Toronto, ON Canada; 13 Department of Ophthalmology, School of Medicine, University of California San Francisco (UCSF), San Francisco, CA USA

Dear Editor,

We read with great interest the paper by Hart de Ruyter et al. [5] in the February issue of *Acta Neuropathologica* assessing the presence of p-tau (total tau (HT7), p-tau Ser202/Thr205, and p-tau Thr217) in the retina in relation to tau pathology in the brain in primary tauopathies and Alzheimer’s disease compared with non-tau-associated pathologies. In tauopathies, several phospho-epitopes of tau were present in the inner plexiform layer (IPL), outer plexiform layer (OPL), and inner nuclear layer (INL). The presence of retinal tau was further correlated with tau depositions in hippocampus and cortical regions. The authors suggest retinal p-tau as a potential biomarker for primary tauopathies. In this letter, we would like to add crucial early imaging data that (1) investigate the structural consequences of tau deposition, (2) support the use of retinal tau markers as a diagnostic tool for tauopathies given their presence early in the course of the disease, and (3) demonstrate the feasibility of these findings outside of pathology studies.

For our investigations, we employed optical coherence tomography (OCT) imaging data from the multi-site *4 Repeat Tauopathy Neuroimaging Initiative (4RTNI)*, which generated a deeply phenotyped cohort including imaging, body fluid samples, and cognitive testing in patients with progressive supranuclear palsy (PSP), corticobasal syndrome (CBS), and healthy controls [10]. OCT is an imaging method using interference of low-coherence light to produce high-resolution cross-sectional retinal images [8]. After reading Hart de Ruyter et al. [5], we decided to quantify thickness changes of the IPL, OPL, and INL in tauopathies compared with healthy controls (HCs) and non-tau-related pathologies in 4RTNI. We also quantified changes in the outer nuclear layer (ONL)—which contains the cell bodies of rod and cone photoreceptors—in line with a recent publication by Arouche-Delaperche that described functional impairment directly due to tau deposition and indirectly due to tau-associated microglial activation in animal models of tauopathy [2]. We further investigated changes in the peripapillary retinal nerve fiber layer (pRNFL) and the ganglion cell layer (GCL), which contain the axons and cell bodies of retinal ganglion cells, respectively, and are of interest as markers of neurodegeneration [4].

OCT acquisition, quality control, and reading were performed centralized at UCSF by experienced technicians. OCT data were acquired with Spectralis SD-OCT devices (Heidelberg Engineering, Heidelberg, Germany) and reported according to APOSTEL recommendations [3]. Image quality was assessed by OSCAR-IB criteria; scans with insufficient image quality (17 eyes from 12 patients (N(PSP) = 10, N(CBS) = 2) were excluded [9]. Semi-automatic intraretinal layer segmentation was performed using the software provided by the OCT manufacturer (Eye Explorer; Heidelberg Engineering). We measured the pRNFL using 12° ring scans around the optic nerve head with activated eye tracker, and all other layers using a 3.45-mm-diameter cylinder around the fovea from a macular volume scan for all subjects (supplementary Fig. 1). Cross-sectional group differences and correlations were explored using linear mixed effect models accounting for within-subject inter-eye correlations as a random effect, as well as controlling for age, sex, and study site (if necessary). Statistical significance was established at *p* < 0.05. Analyses were performed with R 4.1.0.

Our final cohort included 47 patients with PSP and 42 patients with CBS—all were recruited early in their disease course (see Table 1). Eighty-three HCs from a parallel study at the University of California, San Francisco without cognitive impairment were included for comparison. Only the PSP group showed significant thinning of GCL (estimate (B) = − 0.02, SE = 0.01, *p* = 0.03), IPL (B = − 0.02, SE = 0.01, *p* = 0.01), and ONL (B = − 0.05, SE = 0.02, *p* < 0.01) compared with HC (Fig. 1a-c). The thinning of IPL (*p* = 0.04) and ONL (*p* = 0.01) but not GCL was confirmed in a PSP subset with early disease (≤ 5 years since symptom onset, *N* = 34) compared with HC. No differences in other layers in PSP or in CBS compared with HC were noted. In PSP patients early in their disease course, GCL, IPL, and ONL thinning were not correlated with total PSP rating scale scores (PSPRS, [mean (IQR)]: 31 (27, 38)), or with midbrain atrophy and midbrain/pons-ratio quantified on MRI (data not shown).

**Table 1:** Cohort description for patients with PSP, CBS, and HC

	PSP	CBS	HC
Number of patients [N]	47	42	83
Gender [male, N (%)]	22 (47)	22 (52)	41 (49)
Age [years; mean (SD)]	68 (8)	67 (8)	69 (15)
Years since onset [years, mean (IQR)]	4 (3,6)	4 (2,5)	
pRNFL [µm, mean (SD)]	92.8 (11.0)	90.8 (12.6)	90.4 (6.5)
GCL [mm^3^, mean (SD)]	**0.40 (0.06)**	0.41 (0.05)	0.41 (0.04)
IPL [mm^3^, mean (SD)]	**0.34 (0.04)**	0.35 (0.03)	0.36 (0.03)
INL [mm^3^, mean (SD)]	0.32 (0.03)	0.33 (0.03)	0.33 (0.03)
OPL [mm^3^, mean (SD)]	0.30 (0.03)	0.31 (0.03)	0.30 (0.03)
ONL [mm^3^, mean (SD)]	**0.63 (0.10)**	0.67 (0.08)	0.66 (0.07)

Bold metrics are significant compared with HC

*CBS* corticobasal syndrome, *HC* healthy controls, *IQR* inter-quartile range, *N* number, *PSP* progressive supranuclear palsy, *SD* standard deviation

**Fig. 1:**
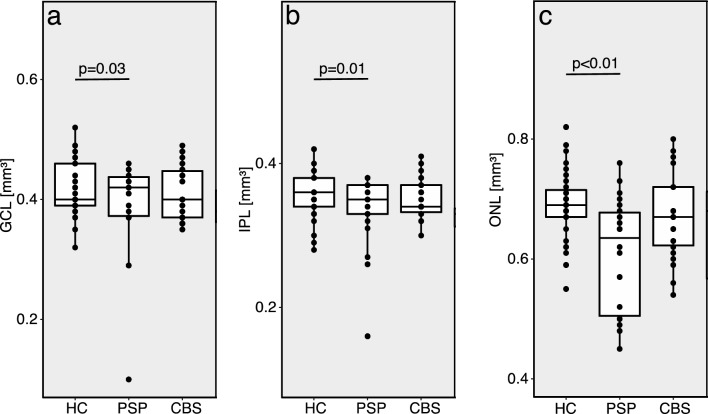
Retinal layer thicknesses in patients with PSP and CBS compared with HC for **a** GCL, **b** IPL and **c** ONL. All parameters are plotted as boxplots with dotted overlay for eye-based values. *CBS* corticobasal syndrome, *GCL* ganglion cell layer, *HC* healthy control, *IPL* inner plexiform layer, *ONL* outer nuclear layer, *p p*-value, *PSP* progressive supranuclear palsy

Ganglion cell loss was not investigated by Hart de Ruyter et al. [5], but was shown recently by Arouche-Delaperche in a tauopathy animal model [2]. The absence of GCL thinning in our subset analysis of patients ≤ 5 years since onset suggests that ganglion cell loss and therefore retinal neurodegeneration occur later in the disease. In contrast, we were able to confirm IPL thinning in PSP patients throughout different time points in the disease course. We did not observe OPL thinning. In Hart de Ruyter et al. [5], a subset of patients showed tau deposition only in the IPL and not in the OPL, whereas other patients had tau deposition in both IPL and OPL. Taken together with our data, this suggests that tau deposition in the IPL might be a relatively early process with potential structural effects such as synaptic loss. To our surprise, we also found ONL thinning in our PSP cohort compared with HCs, despite the absence of ONL tau deposition in the study by Hart de Ruyter et al. [5]. Yet, they described intracellular immunoreactivity for p-tau in the photoreceptor inner segment layer as well as in horizontal and amacrine cells [5]. As suggested in the tau animal model by Arouche-Delaperche et al., these depositions may induce functional deterioration of photoreceptors and lead to atrophy of the cell bodies, ultimately inducing the ONL thinning we observed in PSP.

The absence of the investigated retinal changes in CBS and the consistent changes seen in early PSP suggests that IPL and ONL thickness might be utilizable as biomarkers in PSP. While previous studies were also able to show retinal changes in people with PSP and tau-associated frontotemporal degeneration [1, 6, 7], this study is the first to suggest that the changes in OCT parameters occur very early in the disease course of PSP and might thus be feasible as biomarkers for early diagnosis and prognosis. Yet, it must be kept in mind that distinguishing GCL and IPL as well as ONL and OPL in OCT imaging is a relatively new approach and additional longitudinal monitoring will be necessary to confirm the utility of these markers. The combination with other diagnostic techniques for a multimodal workup might be a valuable approach in the future. Hart de Ruyter et al. [5] also described a predominance of tau deposition in the outer retina, which should be investigated by longitudinal clinical OCT imaging in future studies. However, our data provide a temporal aspect to the pathological data described and support the potential value of retinal changes as a biomarker in primary tauopathies.

### Supplementary Information

Below is the link to the electronic supplementary material.

Supplementary file1 (PDF 672 KB)

## Data Availability

Data cited in the manuscript is available on request in deidentified, aggregated form for preservation of anonymity.
