# Supplementary material for: Scientific commentary on: “Phosphorylated tau in the retina correlates with tau pathology in the brain in Alzheimer’s disease and primary tauopathies”
Source: Acta Neuropathol. 2024 Feb 3;147(1):30. doi: 10.1007/s00401-023-02656-z (PMC10838223; doi:10.1007/s00401-023-02656-z)
Supplement: Supplementary file 1 — Supplementary file1 (PDF 672 KB) [file 401_2023_2656_MOESM1_ESM.pdf]

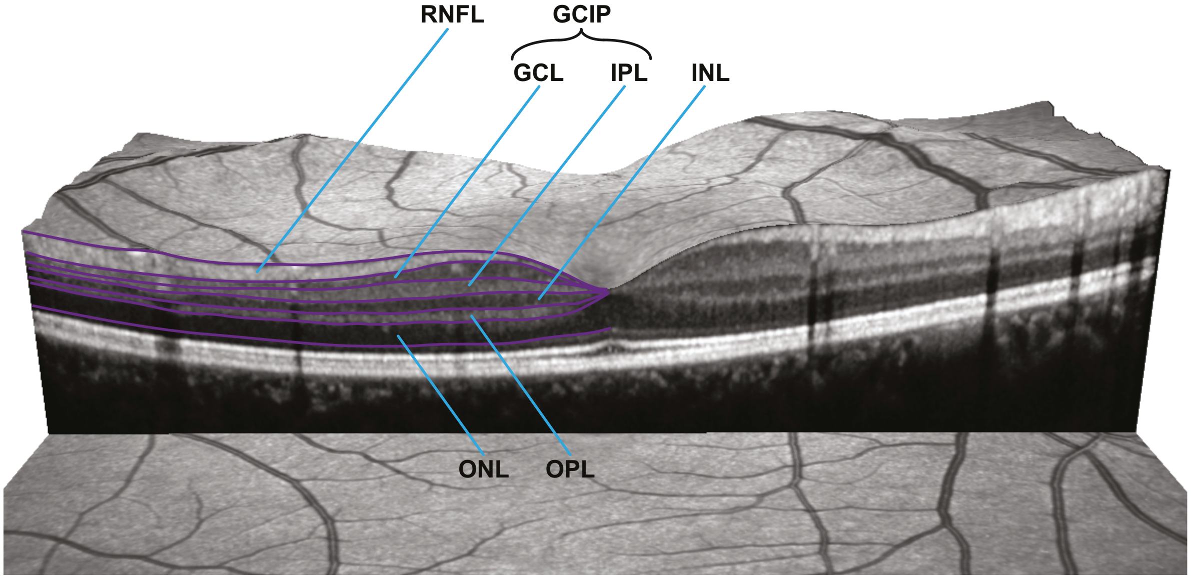

Supplementary figure 1: Schematic figure of analyzed retinal layers. Notes: Reproduced from Schematic Figure-Macular OCT with Intraretinal Layers by Neurodiagnostics Laboratory @ Charité-Universitätsmedizin Berlin, Germany. Available from: http://neurodial.de/2017/08/25/schematic-figure-macular-oct-with-intraretinal-layers/. *Abbreviations*: INL, inner nuclear layer; IPL, inner plexiform layer; GCIPL, combined ganglion cell and inner plexiform layer; GCL, ganglion cell layer; ONL, outer nuclear layer; OPL, outer plexiform layer; RNFL, retinal nerve fiber layer.
